# Impact of oncologist payment method on health care outcomes, costs, quality: a rapid review

**DOI:** 10.1186/s13643-016-0341-2

**Published:** 2016-09-21

**Authors:** Emily McPherson, Lindsay Hedden, Dean A Regier

**Affiliations:** 1 Canadian Centre for Applied Research in Cancer Control (ARCC), School of Population and Public Health, University of British Columbia, 675 West 10th Avenue, Vancouver, British Columbia V5Z 1G1 Canada; 2 Centre for Clinical Epidemiology and Evaluation, University of British Columbia, 828 West 10th Avenue, Vancouver, British Columbia V5Z 1M9 Canada

**Keywords:** Physician reimbursement, Physician payment, Oncology, Fee-for-service, Salary, Capitation, Activity-based funding, Prospective payment, Pay for performance, Payment by results

## Abstract

**Background:**

The incidence of cancer and the cost of its treatment continue to rise. The effect of these dual forces is a major burden on the system of health care financing. One cost containment approach involves changing the way physicians are paid. Payers are testing reimbursement methods such as capitation and prospective payment while also evaluating how the changes impact health outcomes, resource utilization, and quality of care. The purpose of this study is to identify evidence related to physician payment methods’ impacts, with a focus on cancer control.

**Methods:**

We conducted a rapid review. This involved defining eligibility criteria, identifying a search strategy, performing study selection according to the eligibility criteria, and abstracting data from included studies. This process was accompanied by a gray literature search for special topics.

**Results:**

The incentives in fee-for-service payment systems generally lead to health care services being applied inconsistently because providers practice independently with few systems in place for developing treatment protocols and practice reviews. This inconsistency is pronounced in cancer care because much of the total per patient spending occurs in the last month of life. Some insurers are predicting that this variation can be reduced through the use of prospective or bundled payments combined with decision support systems. Workload, recruitment, and retention are all affected by changes to physician payment models; effects seem to be magnified in the specialist context as their several extra years of training lower their overall supply.

**Conclusions:**

Experimentation with physician payment methods has tended to neglect cancer care providers. Policymakers designing cancer-focused physician reimbursement pilot programs should incorporate quality measurement since very ill patients may receive too little treatment when payment models do not cover oncologists’ total costs, e.g., fee-for-service systems whose prices do not account for the possible presence of other diseases.

**Electronic supplementary material:**

The online version of this article (doi:10.1186/s13643-016-0341-2) contains supplementary material, which is available to authorized users.

## Background

Cancer is a leading cause of morbidity and mortality in Canada. In addition to human suffering, cancer annually costs the health system an estimated C$4 billion and is the largest contributor to lost economic productivity [1]. This trend will not abate, with incident cases in British Columbia (BC) expected to increase by 57 % between 2012 and 2030 [2]. Providing high-quality care to patients that is also cost-effective is an ongoing challenge for cancer control. Policymakers face the challenge to control cost as increases in health care spending put pressure on other government priorities such as education and defense. Since labor costs account for 15 % of health care budgets in Canada [3], payers are exploring how they might contain costs by critically evaluating the way physicians are paid and how changes in payment method will affect health outcomes, resource utilization, and quality of care.

Physicians are tasked to deliver care that maximizes patient benefit. Information asymmetry in medical treatment requires principals (e.g., third-party payers; patients) to rely on agents (e.g., physicians) to recommend and communicate the consequences of alternative courses of action [4]. Agents must be incentivized to maximize benefits to patients, rather than solely to the agent’s own benefit. In their most basic form, financial incentives from remuneration are created through transferring money from the principals to the agent to provide care at a specified level of quality. Economic theory suggests that incentives may be used to reduce the marginal cost of physician behavior change, e.g., increasing adherence to evidence-based guidelines [5]. If the size of the monetary incentive is greater than the cost for the physician changing their behavior, the profit (or portion thereof) may be used as a reward to the physician. The magnitude of change and direction of behavioral response (i.e., incentive vs. disincentive) will depend on a number of factors, including the characteristics of the incentive payment method and the financial and opportunity costs of participating in incentive schemes. These factors are important because poorly designed incentives may have unintended behavioral effects and lead to lower levels of quality, e.g., if an overly large payment wrongly signals high risk [6].

### Major payment methods

There are two primary attributes of physician remuneration that influence the magnitude and direction of physician behavior response: method of payment and level of payment [7]. Payment methods include capitation, fee-for-service, performance-based payment, prospective payment, and/or salary. The timing of the payment can be prospective, i.e., set in advance according to a fixed budget, or retrospective with or without a cap on total payments that are made.

The second attribute, payment level, may be fixed in advance or subject to negotiation after care is delivered. Alternatively, physicians may have complete or partial discretion as to the amount of money charged for services. The amount of payment for physician services may be reduced or withheld if behavior does not conform to benefit-maximizing requirements. Finally, the amount may vary depending on characteristics of the provider or patients seen (e.g., more complex cases receive higher payments). Table 1 provides an overview of each category of payment method, including the terms associated with the payment approach, the definition of each category and the potential benefits and harms of the payment approaches.

**Table 1:** Overview of physician payment approaches

Payment model	Definition	Potential benefits and harms
Capitation; pre-payment	Providers are paid a set amount for each person enrolled with them regardless of whether the person receives care.	May reduce unnecessary health services utilization since payment is not tied to service provision. It is argued the financial incentives in capitation will lead primary care physicians to reduce referrals to specialists [12]. However, some argue that providers may be incentivized to develop overly long lists and actually refer to specialist care too frequently [33].
Fee-for-service	Providers are paid separately for all medical services delivered	In this method, providers are reimbursed for all medical services they provide, lowering the risk of taking on patients who need many services. However, appointments may be limited to one service and complicated patients may require many appointments. This method may also increase the use of services which can give diminishing marginal returns or even have detrimental effects [33] and incentivize the over-delivery of care because it rewards increases in service volume, regardless of health benefit [11].
Pay for performance; payment by results; performance-based payment; results-based purchasing; value-based purchasing; target payments	Providers receive different payments for meeting or missing performance benchmarks, e.g., related to quality, efficiency, care integration [8].	Incentives based on achieving quality objectives are expected to be associated with behaviors designed to achieve the quality targets, e.g., immunization rates, mammography screening, patient satisfaction scores [16]. Risk adjustment algorithms should be employed so that organizations are not penalized for treating sicker patients.
Prospective payment; activity-based funding; bundled payment; lump-sum payment; block funding; clinical pathways	A fixed payment for each patient, based only on the patient’s diagnosis	May reduce clinical variation and end-of-life costs [40]. However, without a focus on quality measurement, the pressures of these systems may place perverse incentives on providers to deliver less care [51]. The development of “clinical pathways” (management plans that address quality by providing the sequence and timing of actions covered by the associated lump sum payment [55]) aims to address this issue.
Salary	Individual providers get a fixed fee per year regardless of the number of patients they treat	Similar to capitation, this method may have utilization lowering effects. However, care quality may be compromised if providers respond to fixed payment by working shorter hours and being less responsive to their patients’ needs and demands [56].

In the United States (US), most recent reforms aiming to change the way health care is funded have focused on hospital payment, for example using global budgets, shared savings programs, penalties for readmissions, and hospital-acquired conditions, rather than changing the way providers are paid [8]. However, the 2015 Medicare Access and CHIP Reauthorization Act offers Medicare patients’ physicians a choice of payment models, e.g., participating in the Merit-Based Incentive Payment System, which starting in 2019 will adjust a provider’s fee-for-service reimbursement up or down based on provider performance on quality measures that are currently being developed [9]. In Canada, activity-based funding for hospitals have been implemented in at least three provinces, with most physicians receiving some form of blended payment, followed by fee-for-service [10].

### Payment method impacts

The impacts of provider payment methods have been extensively evaluated. For example, researchers have found some correlation between the fee-for-service payment model and increased use of tests [11]. They note that higher service use does not necessarily improve outcomes and may even be harmful. Changes from fee-for-service to capitated payment models have also been studied several times. This change does not appear to cause problematic decreases in primary care access [12], but does not decrease hospital use [13].

The use of prospective payment may increase the rate of hospital readmissions and adverse events, but this increase is related to hospitals assigning more severe diagnoses to patients under prospective payment than they would under other systems [14]. This means that the hospital is allocated more funding, although direct treatment costs have not increased.

Cash bonuses have been demonstrated to improve some outcomes—vaccination rates, for example—but researchers caution that the improvement observed may be due to better reporting rather than true practice change [15]. The research indicates that combining payment model changes with other interventions such as educational campaigns may be needed to make meaningful practice changes [15].

Provider payment reforms such as accountable care organization (ACO) shared savings programs encourage providers to form groups and assume responsibility for the care of a population of patients in order to share in payer savings if quality and cost performance benchmarks are achieved. However, a 2001 study found that forming these groups had no significant effect on factors such as improved care coordination and innovation. The authors hypothesized that this may be because the groups did not identify as cohesive entities and used the structure mainly for legal purposes rather than to improve care provision [16].

Broadly, existing evidence suggests that changes in physician behaviors have the potential to impact the cost and quality of care provided. Payment method can also influence recruitment and retention of physicians, which in turn impacts patient access and quality of care [17].

### Object of the document

**Fig. 1:**
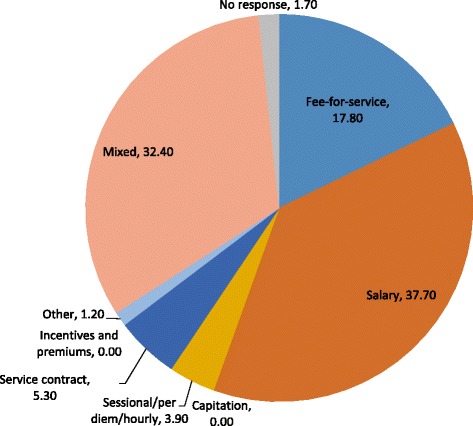
Medical oncologist responses to 2013 National Physicians Survey [22] question 6a on remuneration method (percentage of total)

Previous reviews [18–21] have evaluated the impact of different payment methods on cost and quality of care, but none have focused specifically on the cancer care context. Across Canada, oncology remuneration takes several different forms. Sourced from the 2013 National Physician Survey [22], Fig. 1 shows that salary is the most common remuneration method for medical oncologists (37.7 % of respondents); other methods reported are fee-for-service (17.8 %), “sessional/per diem” (3.9 %), none of these (1.2 %), and a blend of these methods (32.4 %). No oncologists reported payment by capitation or “incentives and premiums”.

The objective of this study is to undertake a rapid review of the literature—a database search combined with a hand search of several systematic reviews and the gray literature—to explore the impact of physician payment methods on patient outcomes, care quality, and overall expenditure, with a specific focus on cancer control in Canada.

## Methods

We followed guidelines for performing a rapid literature review [23], which included defining eligibility criteria, identifying a search strategy, performing study selection according to the eligibility criteria, and abstracting data; this was followed up by an ancillary search for special topics. These searches were conducted in June 2015.

### Eligibility criteria

We set out to include all studies published in English published in the past 10 years, regardless of whether they were original analyses or reviews of past work. We defined “impact” as the consequences of physician payment methods on health services use, expenditures, health outcomes, physician retention, and stakeholder opinion. We excluded abstracts, editorials, letters, and news.

### Search strategies

We worked with a senior librarian at the BC Cancer Agency to help identify subject headings and keywords. We also identified search terms and keywords from key background articles.

Studies were identified through bibliographic searches of the MEDLINE, Embase, and Evidence Based Medicine Reviews publication databases using the following terms and variations upon these:

- Physicians
- Oncology
- Neoplasm
- Activity-based funding
- Prospective payment
- Bundled payment
- Lump-sum payment
- Pay for performance
- Payment by results
- Performance based payment
- Results-based purchasing
- Value-based purchasing
- Target payments
- Capitation
- Pre-payment
- Salary
- Fee-for-service

Under the guidance of the librarian, we undertook an iterative process to customize and refine the search strategy. Table 2 presents the full search strategies for each database. Although the strategies contain statements that emphasize results specific to Canada, these are combined with other statements that use “or”; as such, the search has no regional limitation.

**Table 2:** Search strategies by database

MEDLINE		
1 payment by results.mp.		132
2 activity based funding.mp.		34
3 prospective payment.mp.		2471
4 results based purchasing.mp.		0
5 pay for performance.mp.		1388
6 value based purchasing.mp.		406
7 performance based payment.mp.		32
8 Value-Based Purchasing/		221
9 1 or 2 or 3 or 4 or 5 or 6 or 7 or 8		4376
10 (salar* or cash or funding or remunerat* or reimburs* or capitation).m_titl.		10,331
11 exp reimbursement mechanisms/or exp fee-for-service plans/or exp prospective payment system/		19,943
12 exp “Fees and Charges”/		14,265
13 economics, medical/or fees, medical/or exp Economics, Dental/		4119
14 exp Income/		30,587
15 “costs and cost analysis”/		17,763
16 exp models, economic/		9415
17 9 or 10 or 11 or 12 or 13 or 14 or 15 or 16		91,620
18 Physician Incentive Plans/		1686
19 exp Physicians/ec [Economics]		2901
20 economics, medical/or fees, medical/		3308
21 Physician’s Practice Patterns/		38,532
22 “episode of care”/		1314
23 Patient Care Bundles/		53
24 (physician* adj3 (remunerat* or reimburs* or payment*)).mp. [mp = title, abstract, original title, name of substance word, subject heading word, keyword heading word, protocol supplementary concept word, rare disease supplementary concept word, unique identifier]		1082
25 exp reimbursement mechanisms/or exp fee-for-service plans/or exp prospective payment system/		19,943
26 exp health personnel/ec		8622
27 18 or 19 or 20 or 21 or 22 or 23 or 24 or 25 or 26		70,140
28 exp Neoplasms/		1,465,616
29 medical oncology/or radiation oncology/		12,921
30 exp Antineoplastic Agents/		503,154
31 Cancer Care Facilities/		2772
32 Oncology Nursing/or Oncology Service, Hospital/		6186
33 (cancer* or oncolog* or chemotherap* or radiotherap* or radiation therap*).m_titl.		549,429
34 28 or 29 or 30 or 31 or 32 or 33		1,800,023
35 17 and 27 and 34		983
36 exp Canada/		77,345
37 (canad* or british columbia or alberta or ontario or quebec or manitoba or saskatchewan or nova scotia or new brunswick or newfoundland or prince edward island).mp. [mp = title, abstract, original title, name of substance word, subject heading word, keyword heading word, protocol supplementary concept word, rare disease supplementary concept word, unique identifier]		118,540
38 36 or 37		118,680
39 27 and 34 and 38		285
40 9 and (10 or 11 or 12 or 13 or 14 or 15 or 16) and 27 and 34		47
41 9 and 27 and 34		50
42 9 and 34		99
43 9 and 27 and 38		39
44 9 and 34 and 38		4
45 39 or 40 or 41 or 42 or 43 or 44		419
46 limit 45 to yr = “2005 -Current”		286
47 limit 46 to english language		275
Embase		
Embase <1974 to 2015 May 14>		
#	Search statement	Results
1	payment by results.mp.	253
2	activity based funding.mp.	58
3	prospective payment.mp.	8724
4	results based purchasing.mp.	0
5	pay for performance.mp.	1859
6	value based purchasing.mp.	320
7	performance based payment.mp.	43
8	Value-Based Purchasing/	2810
9	1 or 2 or 3 or 4 or 5 or 6 or 7 or 8	13,742
10	(salar* or cash or funding or remunerat* or reimburs* or capitation).m_titl.	18,835
11	exp reimbursement mechanisms/or exp fee-for-service plans/or exp prospective payment system/	55,981
12	exp “Fees and Charges”/	35,171
13	economics, medical/or fees, medical/or exp Economics, Dental/	651,978
14	exp Income/	71,061
15	“costs and cost analysis”/	53,721
16	exp models, economic/	115,183
17	9 or 10 or 11 or 12 or 13 or 14 or 15 or 16	857,409
18	Physician Incentive Plans/	51,522
19	exp Physicians/ec [Economics]	0
20	economics, medical/or fees, medical/	45,653
21	Physician’s Practice Patterns/	183,699
22	“episode of care”/	209,403
23	Patient Care Bundles/	188
24	(physician* adj3 (remunerat* or reimburs* or payment*)).mp. [mp = title, abstract, subject headings, heading word, drug trade name, original title, device manufacturer, drug manufacturer, device trade name, keyword]	5896
25	exp reimbursement mechanisms/or exp fee-for-service plans/or exp prospective payment system/	55,981
26	exp health personnel/ec	0
27	18 or 19 or 20 or 21 or 22 or 23 or 24 or 25 or 26	506,044
28	exp Neoplasms/	3,504,879
29	medical oncology/or radiation oncology/	108,563
30	exp Antineoplastic Agents/	1,565,429
31	Cancer Care Facilities/	19,458
32	Oncology Nursing/or Oncology Service, Hospital/	25,476
33	(cancer* or oncolog* or chemotherap* or radiotherap* or radiation therap*).m_titl.	1,058,603
34	28 or 29 or 30 or 31 or 32 or 33	4,403,463
35	17 and 27 and 34	12,766
36	exp Canada/	136,398
37	(canad* or british columbia or alberta or ontario or quebec or manitoba or saskatchewan or nova scotia or new brunswick or newfoundland or prince edward island).mp. [mp = title, abstract, subject headings, heading word, drug trade name, original title, device manufacturer, drug manufacturer, device trade name, keyword]	228,077
38	36 or 37	228,077
39	27 and 34 and 38	1648
40	9 and (10 or 11 or 12 or 13 or 14 or 15 or 16) and 27 and 34	367
41	9 and 27 and 34	382
42	9 and 34	554
43	9 and 27 and 38	118
44	9 and 34 and 38	14
45	39 or 40 or 41 or 42 or 43 or 44	2304
46	limit 45 to yr = “2005 -Current”	1833
47	limit 46 to english language	1788
48	“health policy economics and management”.ec.	504,366
49	47 and 48	416
50	(physician* and (fee or fees or pay* or remunerat* or compensat* or purchas* or reimburs*)).m_titl.	2372
51	limit 50 to (english language and yr = “2005 -Current”)	694
52	49 or 51	1104
53	limit 52 to yr = “2013 -Current”	291
54	47 and physician*.mp. and (fee or fees or pay* or remunerat* or compensat* or purchas* or reimburs*).mp. [mp = title, abstract, subject headings, heading word, drug trade name, original title, device manufacturer, drug manufacturer, device trade name, keyword]	151
55	52 or 54	1177
56	limit 55 to exclude medline journals	85
57	53 or 56	352
58	remove duplicates from 57	342
EBM Reviews		
EBM Reviews - Cochrane Database of Systematic Reviews <2005 to December 2014>		
#	Search statement	Results
1	(Cochrane Effective Practice and Organisation of Care Group).mp. [mp = title, short title, abstract, full text, keywords, caption text]	91
2	1 and (canad* or british columbia).ti, kw.	0
3	1 and (cancer* or oncolog* or neoplasms or tumor* or tumour* or chronic).ti,kw.	3
4	1 and (physician* or specialit* or specialt* or dentist* or cost* or financ* or econom* or fees or reimburs* or pay* or salar* or remunerat* or fund* or cash or incentive*or bundle* or performance or capitation or pattern* or episode*).ti, kw.	25
5	remunerat*.ti, kw.	1
6	3 or 4 or 5	28

### Gray literature search

We also undertook a gray literature search. This was limited to oncology in Canada for time and scope reasons. We searched abstracts contained in the Canadian Health Human Resources Network online library [24], as well as websites for the following organizations:

- Canadian Association of Medical Oncologists [25]
- Canadian Association of Radiation Oncologists [26]
- Canadian Foundation for Healthcare Improvement [27]
- Canadian Institute for Health Information [28]
- Institute for Clinical Evaluative Sciences [29]
- National Physicians Survey [22]
- Statistics Canada [30]

### Study selection and data abstraction

One reviewer conducted screening. Initially, we reviewed article titles and abstracts for relevance; the full text of articles that appeared to be potentially eligible were subsequently reviewed for inclusion. EM abstracted the following data from each included article: authors, publication date, country, title, payment approach, health issue, outcomes measured, research methods, and study findings. We worked closely with a senior librarian who helped design and calibrate the search strategies presented in full in Table 2. In-duplicate data extraction and post hoc data extraction review were not performed. We encourage future reviews of this literature body that employ full systematic review methods to include these activities.

### Study acquisition flow

**Fig. 2:**
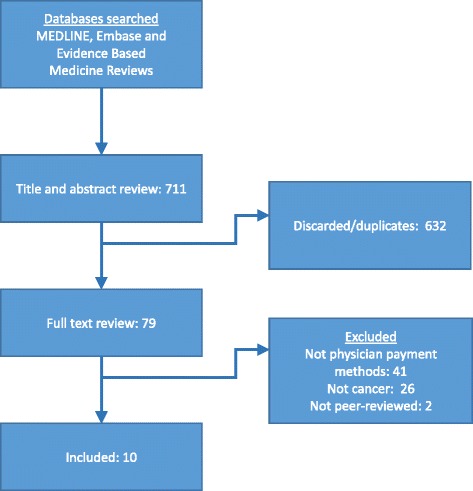
Study acquisition flow from database search

Figure 2 presents the acquisition flow of included studies from the database search. Of 711 citations identified by the database search, ten addressed the impact of physician payment methods on quality or access to care, equity, cost, or efficiency in the context of oncology. Although we are specifically interested in the effect of payment methods in the Canadian context, the relative paucity of studies encouraged us to include research conducted outside Canada. Barring major contextual differences, payment method effects should be similar across jurisdictions. Additional file 1 contains our populated PRISMA checklist.

## Results

This section reviews the findings of the articles returned by the database search and the ancillary search. A subsection highlights the research methods used to evaluate the consequences of physician payment methods.

### Study characteristics

Table 3 presents the key characteristics of the database search articles according to geographical location of study, study design, sampling method, and sample size. The majority of included articles were from the US and three studies were from Canada. Study designs included a mix of quantitative and qualitative approaches, including observational studies (using administrative databases) [6, 12–14, 31, 32], literature reviews [11, 33–36], semi-structured interviews [37], and collection survey [15, 38–40]. Table 4 shows which payment methods each article discusses.

**Table 3:** Characteristics of the identified articles from the database search

Characteristic	Number of articles
Geographic location	
Canada	2
China	1
Denmark	0
Norway	0
South Africa	0
USA	7
Study design	
Literature review/commentary	4
Qualitative survey/interviews	3
Statistical analysis	3 (regression analysis)
Sampling method	
Random	0
All who agreed to participate and were eligible	6
Not applicable	4
Sample size	
<100	3
100 < *n* < 1000	1
>1000	2
Not applicable	4

**Table 4:**
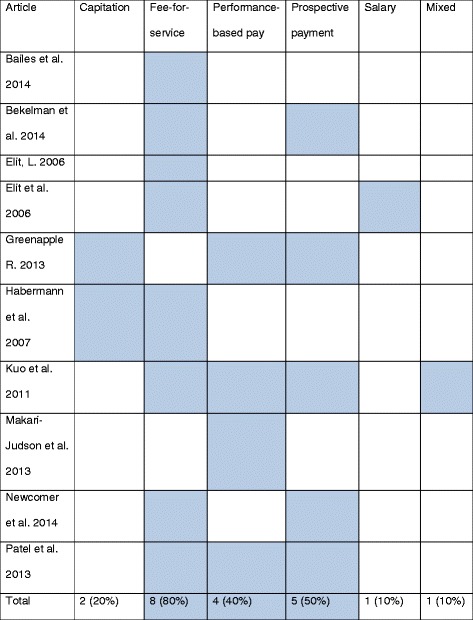
Payment methods discussed

The shaded area indicates that the article in a given row discusses the payment method listed in the corresponding column header

### Articles identified from the database search

Table 5 summarizes the key attributes of the ten articles identified through the database search examining physician remuneration in oncology. Four articles [11, 35, 36, 41] are based on literature reviews or commentary. As such, the findings may not be unique to the article.

**Table 5:** Database search articles

Authors	Title	Payment approach	Methods	Health issue	Outcomes measured	Findings
Bailes JS and Coleman TS. 2014 (USA)	The long battle over payment for oncology services in the office setting [35]	Fee-for-service	Reviews Medicare policy history and reports expert opinion	Outpatient Chemotherapy	Physician fees for chemotherapy drugs	Payments for drug administration can be much less than its cost. Marginal revenue from drug payments is used to make up the difference, and drug payment decreases could result in provider losses.
Bekelman JE, Epstein AJ and Emanuel EJ. 2014 (USA)	Getting the next version of payment policy “right” on the road toward accountable cancer care [11]	Fee-for-service vs. prospective payment	Reviews published literature and agency documentation	Cancer care	Changes in costs and outcomes	Prospective payment systems should include performance measurement to counter associated perverse incentives. For complex cases lump sum payment could be combined with fee-for-service.
Elit, L. 2006 (Canada)	An analysis of alternative funding for physicians practicing gynecologic oncology in Ontario, Canada prior to 2001 [36]	Fee-for-service	Literature search, discussion with stakeholders, meeting minutes from groups considering alternate funding systems	Gynecologic cancer	Events preceding reform of the funding agreement with gynecologic oncologists	Fee-for-service does not account for the increased complexity of services on cancer patients, causing losses and making recruitment and retention difficult.
Elit L, Cosby J and Gynecologic Oncology Group in Ontario. 2006 (Canada)	Does shifting a physician payment system shift physician priorities? A multi-site evaluation of an alternative payment plan (APP) for gynecologic oncologists in Ontario [37]	Fee-for-service vs. a negotiated arrangement where contracts are made with physician groups who are paid a fixed amount regardless of productivity	Interviews with 14 Ontario gynecologic oncologists; interviews were analyzed using grounded theory.	Gynecologic cancer	Changes in physician behavior in response to the new payment system	The new plan improved quality of life and income predictability, increased preventive health care work. Vacancies were filled and staff were retained. Staff delegated follow-up with less complicated patients. The plan did not reduce workload.
Greenapple R. 2013 (USA)	Rapid expansion of new oncology care delivery payment models: results from a payer survey [40]	Comparing “clinical pathways” (bundled payments with quality management), capitation, shared savings and pay-for-performance	A validated survey of payers representing more than 100 million individuals that asked payers about models of care that could improve quality and reduce costs.	Cancer care	Payer perceptions of which payment models are most effective	Payers believe that clinical pathways can reduce clinical variation in care, improve quality and reduce costs, mainly by reducing end-of-life costs
Habermann EB, Virnig BA, Riley GF, and Baxter NN. 2007 (USA)	The Impact of a Change in Medicare Reimbursement Policy and HEDIS Measures on Stage at Diagnosis Among Medicare HMO and Fee-For-Service Female Breast Cancer Patients [31]	Fee-for-service vs. health maintenance organization (capitation)	Compares the effect of change from biennial to annual mammograms by payment method.	Breast cancer	Surveillance Epidemiology and End Results, Medicare claims database	Women enrolled in the health maintenance organization were more likely than those in fee-for-service to be diagnosed early both before and after the, but after the change, the disparity shrank from 4.7 to 2.3 %.
Kuo RN, Chung KP and Lai MS. 2011 (China)	Effect of the pay-for-performance program for breast cancer care in Taiwan [40]	Fee-for-service/activity-based funding vs. pay-for-performance (encouraging evidence-based therapy and reward better patient outcomes)	A retrospective analysis of patients who received curative surgery. Multivariate regression analyzed the association between program enrollment and quality of care.	Breast cancer	Population-based cancer registration and claims data	Enrollees received higher-quality care, had better 5-year overall survival and less recurrence
Makari-Judson G, Wrenn T, Mertens WC, Josephson G and Stewart JA. 2014 (USA)	Using Quality Oncology Practice Initiative Metrics for Physician Incentive Compensation	Pay for performance	Based on their performance in five achievement categories, physicians were offered a bonus percentage of salary corresponding to the target level achieved.	Hematology oncology	Work relative value units, Quality Oncology Practice Initiative metrics, patient emotional well-being from medical records, academic goals and the overall financial success of the group	Results are reported for two measures: quality and emotional well-being. For the former, “Tier III” was achieved resulting in a bonus of 24 % salary. For the latter no bonus was achieved.
Newcomer LN, Gould B, Page RD, Donelan SA and Perkins M. 2014 (USA)	Changing Physician Incentives for Affordable, Quality Cancer Care: Results of an Episode Payment Model	Fee-for-service vs. episode payments (bundled payments)	Physicians at five medical oncology groups were reimbursed with a single episode payment for services to cancer patients as part of a pilot program. The episode cohort was compared with a control fee-for-service cohort.	Breast, colon and lung cancer	Clinical data corresponding to characteristics of episode payments (cancer type, stage, genetic profile), claims data, average chemotherapy drug sale price	The total medical cost for the episode cohort was $33.4 million less than what was predicted using fee-for-service.
Patel KK, Morin AJ, Nadel JL and McClellan MB. 2013 (USA)	Meaningful Physician Payment Reform in Oncology	Clinical pathways (bundled payments), pay for performance, fee-for-service	Reviews pilot initiatives in the US that combine physician payment reforms with delivery reforms.	Cancer care	Research on oncology practice and the impact of physician payment methods, proposals from oncology societies	The authors propose a payment model that combines fee-for service payment with case management payment and a care coordination fee, increasing total provider payment but potentially decreasing the total care cost.

Habermann et al. [31] used data from the Medicare cancer registry (part of the National Cancer Institute’s Surveillance Epidemiology and End Results project) to compare breast cancer screening rates in health maintenance organizations (HMOs) with rates for providers reimbursed by fee-for-service. (Physicians practicing in HMOs are normally reimbursed through capitation [42]). Health maintenance organizations and managed care generally tend to use more resources at the beginning of the care process, e.g., performing preventive care, in order to keep people healthier and also save money in the long run [43]. Cancer stage at diagnosis, a proxy for screening rate, was estimated using a logistic regression model that adjusted for payment method as well as demographic variables. The authors found that women enrolled in health maintenance organizations were more likely than those in fee-for-service to be diagnosed early (and therefore likely to have received screening), both before and after a change from biennial to annual mammograms. However, this difference decreased by half after the move to annual screening.

Elit [36] analyzed the events that led to a change in payment method for Ontario-based gynecologic oncologists by conducting a non-systematic search of academic and gray literature and also speaking to key stakeholders including university physicians and members of the Ontario Medical Association. She found that most of the province’s gynecologic oncologists changed from fee-for-service remuneration to the salary-based program offered in 2001 because they reported that prices in Ontario’s fee-for-service payment model did not account for the fact that non-oncology procedures are often more costly when performed on cancer patients. Study participants noted that the situation had encouraged specialists to focus on uncomplicated cases. Under-reimbursement had also hampered the recruitment and retention of specialized staff, but retention was improved when the alternative program of salary payment was offered.

In a related study, Elit and Cosby [37] conducted qualitative interviews which explored the impact on gynecologic oncologists of switching from fee-for-service to the alternative salary-based plan described above. They recruited 14 gynecologic oncologists from five practice sites in Ontario, four of which had opted for the change from fee-for-service to salary. A semi-structured interview guide designed for the study was used in the interviews; it consisted of professional and personal questions. The interviewed physicians who experienced the remuneration change reported improvements in their own quality of life and income predictability, while also noting that their preventive care work had increased. They noted that practice site vacancies were able to be filled and staff were able to be retained. It had been hoped that the new program would also reduce physician workload, but the interviewees stated this had not occurred. However, interviewees affirmed that follow-up for less complicated patients was now being appropriately delegated.

Newcomer et al. [44] describe a pilot project where physicians at five medical oncology groups in the US were reimbursed with a single episode payment for all breast, colon, and lung cancer patients at their initial visit. All other physician services continued to be reimbursed via the existing fee-for-service contract. The study design compared the operational and control cohorts during the pre-pilot and pilot time periods. In the analysis, 810 patients were used. Data included clinical data corresponding to characteristic of episode payments (cancer type, stage, genetic profile), the total medical cost per episode of care (a linear regression function of the episode payment condition, age, and sex), and chemotherapy drug cost (average sale price). Controls were obtained from UnitedHealthcare’s registry of more than 65,000 breast, colon, and lung cancer patients. The net savings in total medical cost for the episode cohort compared to fee-for-service was $33.4 million. Although the program contained several incentives to lower drug costs, chemotherapy drug spending unexpectedly rose; it totaled $13.5 million more than predicted at $21 million. The authors state the study was not sufficiently powered to analyze which expenses disproportionately impacted the differences in total medical cost.

Offering expert commentary on the Medicare program’s reimbursement for chemotherapy services, Bailes and Coleman [35] argue that Medicare’s fee-for-service payment system has tended to underestimate the total cost of chemotherapy treatment. The authors state that reimbursement for products and services used in the administration of chemotherapy drugs has often been substantially less than their true cost. To cover the administration resource shortfall, they note that oncologists have relied on marginal profit from drug reimbursement [45]. Decreases in drug payments brought in by the 2003 Medicare Modernization Act have resulted in losses for some oncologists. However, they note that the 2010 Affordable Care Act includes funding for pilot programs to “align nationally recognized, evidence-based guidelines of cancer care with payment incentives … in the areas of treatment planning and follow-up care planning” [46].

Turning to pay for performance, Kuo et al. [32] conducted a retrospective analysis of breast cancer care examining a program targeted at hospitals which rewards better patient outcomes with a bundled payment, which encompasses treatment options based on recommended treatment plan for the breast cancer stage. This payment is higher than in the original payment scheme (case-based for surgery and fee-for-service for other treatment components) when the treatment plan is followed, lower when it is not. The authors note that attending physicians in Taiwan are mainly employed by hospitals, so financial incentives applied at the hospital level may still directly impact physician behavior. Data came from the Taiwan Cancer Database. Women diagnosed in 2003 or 2004 with stage I or II breast cancer were included. The association of program participation and quality of care was estimated using linear regression and controlling for age, stage, comorbidity, and type of surgery. Results showed that patients treated at hospitals participating in the pay-for-performance program received higher-quality care, achieved better 5-year overall survival, and experienced less recurrence [30].

Makari-Judson et al. [47] document the experience of a group of 11 hematologic oncologists who were offered performance-based incentives in five categories (with associated outcome in parentheses): patient-centered goals (measured by the patients’ medical record), quality measures (Quality Oncology Practice Initiative metrics), clinical productivity (work relative value units), academic (not specified), and the group’s overall financial performance (not specified). Incentives were arranged in three tiers; each corresponded to a category score and triggered a bonus (percentage of salary). The authors report results for two of the five measures: patient-centered goals and quality measures. For the latter, “Tier III” was achieved resulting in a bonus of 24 %. For the former, no bonus was achieved.

Greenapple [40] conducted an online survey of 49 American health insurers, representing more than 100 million covered individuals, which asked them about the models of care that they are implementing or would support in order to improve cancer care quality and also control cost. The survey results reveal that the payers most favored systems of “clinical pathways”, a specialized form of care bundle where an evidence-based algorithm guides care practice for a defined group of patients during a set period of time [45]. They believe that these could reduce both the cost of end-of-life care and clinical variation in care, while also improving care quality. The payers prioritize controlling costs through the method of reducing wasteful and inappropriate care and believe that clinical pathways are most likely to achieve such reductions.

In an expert commentary, Patel et al. [41] review pilot initiatives in the US that combine oncologist payment reforms with delivery reforms, including performance incentives, bundled payment and clinical pathways, and mixed methods, i.e., a fee-for-service chemotherapy payment for the cost of buying the drug, fixed payments for drug administration and care management. They propose a physician payment model for cancer care that combines fee-for-service payment with case management payment (to lower the incentive to increase the volume and intensity of patient services) and a care coordination fee. This would involve increasing total payment to physicians but could decrease the total cost of cancer care (by decreasing waste and inefficiency, as well as payments for all other cancer care).

Bekelman et al. [11] use academic literature and public-sector publications to make evidence-based recommendations on reforms to cancer care payment policy. They argue that any prospective payment systems should focus on performance measurement, since theory predicts that lump sum payment systems will place perverse incentives on providers, e.g., providing too few services to very ill patients [6]. The authors recommend a strategy of cross-subsidizing with fee-for-service complex cases treated under prospective payment in order to mitigate the risk to providers of having their total costs exceed the lump payment [11].

### Research methods used to evaluate consequences of physician payment methods

Regression analysis was used by two articles in the database search. One used a pretest-posttest study design [31], where one group is assessed at different time points. The other used a retrospective cohort analysis [32] where two groups are compared at the same time.

Qualitative research methods were employed by two articles [37, 40]. Their study designs involved using literature reviews to inform a semi-structured interview guide or surveying payers and conducting in-person interviews with providers. To analyze the qualitative results, Greenapple [40] calculated the percentage breakdown of participant responses. Two investigators independently analyzed coded data in Elit and Cosby’s study [37]. Then together they discussed themes and developed theory; the model that emerged from the discussion was validated with two final interviews.

Regarding the creditability of study results in general: the studies tended to be exploratory and not designed to provide generalizeable results, but more than two thirds of the articles performed at least some uncertainty analysis of their results.

### Data sources used to evaluate consequences of physician payment methods

Since the provider compensation method was often used at the outset as an indicator dividing the sample into cohorts or “treatment groups”, studies seldom included payment method as an exogenous variable. Instead study authors compared payment method cohorts using data on utilization and expenditures [32] and on patient outcomes and stakeholder opinion (collected through in-person interviews and online surveys) [37, 40].

### Results from the gray literature search

Multiple sources provide information on the ways that oncologists are paid in Canada. However, they do not investigate the outcomes associated with the different payments [10, 22, 29]. For example, the Canadian Institute for Health Information’s (CIHI) National Physician Database 2012–2013 [10] includes payment information for medical specialists, e.g., Fee-for-Service Clinical Payments to Physicians by Province/Territory; oncology is one of the specialties that comprise the medical specialist category.

## Discussion

This review presents the literature’s key findings related to the impact of different physician payment methods and uncovers articles that examine those impacts in the context of cancer care. This is important since the study findings show that payment method impacts on cancer care can run contrary to what would be expected in other disease areas. For example, although Ellis [6] showed that providers reimbursed via fee-for-service are unlikely to discriminate for patient illness severity, Elit [36] saw that oncologists reimbursed through fee-for-service methods were substituting toward patients with less complicated conditions since the fee levels were not sufficient for treating cancer patients.

The Medicare program has been a leader in experimenting with models of physician payment, but it has not focused on the area of cancer care [48]. Indeed this review appears to be one of the first treating the impact of physician payment methods on cancer care. Its findings emphasize the importance of further study of the impacts of changing payment methods for the physicians who focus on cancer care. The gray literature search shows that the CIHI National Physician Database includes oncologist remuneration information by province in Canada and could be used as data source for future projects.

Cancer care tends to include high costs concentrated at the end of life, when relatively low-cost palliative care may be a more effective option, both financially and with regard to the patient’s quality of life. It is estimated that the Medicare program spends one third of the cost of treating cancer in the final year of a patient’s life and 78 % of that spending occurs in the final month [49]. Broomberg [33] argues that this is an expected result of incentives in fee-for-service payment systems, which reinforce doctors’ tendency to apply health care resources inconsistently as they practice independently with few systems in place for developing treatment protocols and practice reviews. It follows that the payers queried in Greenapple’s [40] survey believe that replacing individual services with effective “bundles” of oncology care could bring down end-of-life costs while also improving quality and reducing regional variation.

Workload, recruitment, and retention are all affected by changes to physician payment models. Effects seem to be magnified in the specialist context as their several extra years of training lowers their supply to the system. Payment models that lead to poor retention of providers, for example, if remuneration does not cover physician costs as in the case of chemotherapy drug administration services [35], may lead to heavy workloads which in turn complicate recruitment. Also it has been noted that the proportion of patients assigned “high-severity” status for accounting purposes can be significantly reduced when the workload of the discharging physician is increased, resulting in a substantial revenue loss for the hospital [50]. However, few of the studies that focused on the specialist context examined workforce factors such as these as outcomes [37]. Clearly, future studies aiming to evaluate the impact of changes to payment methods for oncologists in Canada should include measurement and analysis of changes in workload and workforce factors. Levels of hospital utilization and expenditures/claims are obvious starting points in areas where oncologists are primarily employed by hospitals. Yet in addition to these general indicators, payers may also be interested in changes in the likelihood of adverse events since these can have direct, predictable impacts on utilization and expenditure; hospital readmission rates could be used to proxy for adverse events. In terms of office-based oncologists, changes in the number of tests ordered after patient consultations could be examined. In both settings, conformity with guidelines for evidence-based medicine is a way to evaluate the impact of pay-for-performance payment systems.

It is also crucial to evaluate how much of the variation observed after a new payment method’s implementation should be attributed to factors other than that method. A few studies took up this challenge. Kristiansen et al. [39] report that the variables used in their analyses explain only 10 % of the observed variation in laboratory utilization; as a result, they note that the main determinant of test ordering behavior is probably the medical condition. This emphasizes the necessity of conducting studies in the cancer context: to discover factors that drive cost, but can be changed while controlling for unchanging disease complexity. It should also be noted that two studies [14, 38] used random sampling while others targeted a specific population and then included all or most members of the population who agreed to participate.

Many payers are experimenting with different payment strategies. However, some study results may not be generalizeable to other contexts, either because of the research methods or also the structure of the health system studied. An example of structural difference is the Taiwanese health system where doctors (including those at the primary care level) are almost all employed by hospitals [32]. Reforms instituted at the hospital level may broadly affect physician behavior, but this will not be the case in systems where doctors practice independently. Another example is in single payer context where payment models may need to include specific reimbursement for teaching and research services [36]. In multi-payer systems, having several funding streams may make these activities more likely to be funded by one of the payers. As experimentation continues, we should remember that most studies report short-term effects of payment system changes, but the longer term-associated changes in technology use and practice structure may be much larger in magnitude [51]. For example Finkelstein [52] has shown that the implementation of Medicare in 1965, which caused a large increase in fee-for-service reimbursement, led to much larger effects on cost, technology use, and practice delivery over time than was suggested by initial, static analyses.

This review experiences some methodological limitations common to rapid literature reviews. The lack of a systematic review process implies some study selection bias. Although we evaluate the credibility of study findings, we did not include a systematic quality assessment process; it has been argued that forgoing such a process is a source of bias in rapid reviews [53]. However, the body of literature was so small that eliminating work based on a quality rubric would likely have left us too little to review. Another possible limitation is that we did not perform in-duplicate data extraction and post hoc data extraction review. Further, in the current policy environment, where as noted above the MACRA legislation is overhauling physician payment methods in the huge system of US Medicare, rapid reviews have an advantage over systematic reviews because they can be produced quickly to facilitate evidence-based policymaking.

The review includes only articles published in English. While limiting results in this way could omit Quebec-specific results, prior research [54] shows that English-language-restricted literature searches tend to have similar results to those without language restrictions (when the review’s content is mainly based within published literature). A follow-up gray literature search could be expanded to include French content.

## Conclusions

This review presents currently published literature related to the impact physician payment method has on cancer care. It shows that, although general impacts of physician payment methods have been well-studied, research has seldom been extended to the specialized circumstances of cancer care.

Nevertheless, several findings have implications for decision-makers concerned with the impact of physician payment systems on cancer care. Patients with high-severity illnesses may receive too little treatment in bundled payment systems that rely on patient diagnosis so it may be prudent to invest in quality measurement programs when implementing these systems. Fee-for-service payment models can also lead to too little treatment when prices do not vary to account for patient status, e.g., when a treatment is not complex in principle, but it is made so by the overall poor health of the patient. However, we see that even high levels of treatment are not necessarily a corollary for quality of care, so even when fee-for-service incentivizes the provision of too much treatment, it may still need to incorporate quality measurement.

Cancer care is resource-intensive: technologies are expensive and treatments are time-intensive. The time is right to evaluate outcomes that occur before and after reforms to oncologist payment methods, for example, BC’s recent move from fee-for-service to salary-based payment. Others could leverage the results of this experiment to avoid costly duplication. Recruitment and retention rates should also be examined to further quantify impacts of new programs; effects on research production and teaching programs are also of interest.

## Additional file

Additional file 1: PRISMA checklist. (PDF 218 kb)

## Abbreviations

BC: British Columbia
CIHI: Canadian Institute for Health Information
HMO: Health maintenance organizations
US: United States
